# Heart and Brain: Linking Obesity-Related Inflammation, Cardiovascular Disease and Cognitive Function

**DOI:** 10.3390/jcdd13100477

**Published:** 2026-09-22

**Authors:** Allison B. Reiss, Shelly Gulkarov, Umar Syed, Gabriel Ben-Dor, Ankita Srivastava, Milanjit Singh, Joshua De Leon

**Affiliations:** 1 Department of Medicine, NYU Grossman Long Island School of Medicine, Mineola, NY 11501, USA; joshua.deleon@nyulangone.org; 2 Department of Foundations of Medicine, NYU Grossman Long Island School of Medicine, Mineola, NY 11501, USA; shellygulk1234@gmail.com (S.G.); umar.syed@nyulangone.org (U.S.); ankita.srivastava@nyulangone.org (A.S.); milanjitsingh8522@gmail.com (M.S.); 3 Departments of Neurology and Psychiatry, NYU Grossman Long Island School of Medicine, Mineola, NY 11501, USA; gabriel.ben-dor@nyulangone.org

**Keywords:** heart–brain axis, cardiometabolic health, obesity, type 2 diabetes mellitus oxidative stress, endothelial dysfunction, insulin resistance, dyslipidemia, hypertension

## Abstract

The heart and brain are interconnected, and risk factors that impact the heart can negatively affect the brain and cognition. Among the underlying features that foster both poor brain health and poor heart health are obesity, diabetes, physical inactivity and low sleep quality. Obesity and type 2 diabetes are associated with an increased likelihood of cognitive impairment and dementia. Further, obesity is a causal factor in obstructive sleep apnea, leading to intermittent hypoxemia, neuroinflammation and cognitive dysfunction. Cerebrovascular disease with reduced cerebral blood flow brings on stroke and elevates dementia risk. Like cardiovascular disease, cerebrovascular disease is also accelerated in the setting of obesity, likely through the same mechanisms involving endothelial dysfunction and damage to macrovasculature and microvasculature from hypertension, dyslipidemia and inflammation. Obesity is considered a risk factor for recurrent stroke. The protracted low-grade chronic elevation of inflammatory markers found with excess adiposity leads to oxidative stress and promotes both endothelial dysfunction and a pro-thrombotic state. Greater loss of brain volume has been documented in middle-aged and older adults with obesity compared with young adults with obesity. Obesity poses a threat to the cardiovascular and cerebrovascular health and well-being of individuals worldwide. Unfortunately, treatments directed at reducing caloric intake are of limited effectiveness. This review will focus on the latest research into the link between excess adipose tissue and cardiac and brain health, with attention to dietary and lifestyle interventions that may be protective of the integrity and function of the vasculature and brain.

## 1. Introduction

Obesity is one of the most pressing public health problems worldwide. Its prevalence has risen substantially during the past three decades, and over one billion children, adolescents, and adults are now living with obesity [1]. Obesity is not simply a disorder of excess body weight. It is a chronic disease characterized by altered adipose tissue function, metabolic stress, vascular injury, and chronic low-grade inflammation. These abnormalities contribute to a plethora of comorbidities including type 2 diabetes, hypertension, dyslipidemia, obstructive sleep apnea, cardiovascular disease, stroke, and cognitive impairment [2].

The heart and brain are closely interconnected. Risk factors that injure the cardiovascular system can also impair brain structure and function. Hypertension, diabetes, physical inactivity, obesity, elevated LDL cholesterol, smoking, depression, social isolation, sleep disturbance, and air pollution have all been identified as potentially modifiable contributors to dementia risk [3]. Many of these risk factors cluster in persons with excess adiposity. Thus, obesity provides an important clinical model for understanding how systemic metabolic disease can influence both cardiovascular and cognitive outcomes.

Adipose tissue is now recognized as an endocrine and immunologic organ rather than an inert site of energy storage. In obesity, adipocytes enlarge, visceral and ectopic fat depots expand, and adipose tissue becomes infiltrated by immune cells. These changes promote the release of pro-inflammatory cytokines, free fatty acids, and altered adipokines, contributing to insulin resistance, oxidative stress, endothelial dysfunction, and vascular disease [4,5]. This chronic inflammatory state is a central mechanism linking obesity to disease in organs distant from adipose tissue, including the heart, vasculature, and brain.

This review synthesizes current evidence on the relationships between excess adiposity, cardiovascular dysfunction, cerebrovascular injury, and cognitive decline, with an emphasis on shared and convergent mechanistic pathways and translational implications.

## 2. Methodology

This review was conducted using the transparent, structured narrative literature review approach rather than a formal systematic review design. The peer-reviewed literature published between 2012 and 2026 was identified through a comprehensive literature search in the following electronic databases and academic search platforms: PubMed/MEDLINE, Google Scholar, Embase, Scopus and Web of Science. Search terms used in various combinations included “cardiometabolic disease”, “cardiovascular disease”, “cerebrovascular disease”, “obesity”, “type 2 diabetes”, “stroke”, “cognitive impairment”, “dementia”, “neuroimaging”, “biomarkers”, “obstructive sleep apnea”, “inflammation”, “oxidative stress”, “pathways”, “signaling”, “endothelial dysfunction”, “lifestyle”, and “treatment”. Eligible publications included human observational studies, randomized controlled trials, systematic reviews, meta-analyses, clinical guidelines, and selected translational studies that provided mechanistic context. Priority was given to recent, high-quality studies and landmark investigations with clinically relevant cardiovascular, cerebrovascular, and cognitive outcomes. Seminal studies published before 2012 were included selectively when they provided foundational mechanistic, clinical, or methodological information. The evidence was synthesized qualitatively into thematic sections: obesity-related vascular and metabolic injury, inflammation and endothelial dysfunction, stroke and cerebral hypoperfusion, sleep apnea, structural brain changes, and lifestyle-based strategies for risk reduction. The literature was summarized to highlight the principal mechanistic, epidemiologic, and clinical evidence linking obesity with cardiovascular, cerebrovascular, and cognitive outcomes, while also identifying inconsistencies, knowledge gaps, and remaining uncertainties.

## 3. Obesity as a Chronic Inflammatory State

### 3.1. Adipose Tissue Dysfunction and Release of Pro-Inflammatory Cytokines

Adipose tissue, primarily composed of adipocytes, is a major endocrine and immunoregulatory organ that plays an essential role in glucose and lipid homeostasis [6,7]. Obesity is associated with tissue remodeling and increased fat mass achieved by hypertrophy (increase in adipocyte size) and hyperplasia (increase in adipocyte number) [8]. Excess accumulation of fat in adipocytes leads to obesity and further to insulin resistance [9,10,11]. Adipose tissue accumulation and dysfunction are foremost among drivers of the pathophysiological processes leading to obesity-related metabolic disorders. Obesity and adipose tissue dysfunction are the outcome of a combination of factors such as chronic nutrient excess, physical inactivity and genetic predisposition. The cellular changes associated with adipose tissue dysfunction include adipocyte hypertrophy, ectopic fat accumulation, hypoxia and a chronic inflammatory response [12,13,14,15]. Increased adipose tissue mass can exceed the capacity of capillaries to deliver oxygen, resulting in an oxygen deficit and tissue hypoxia [16,17,18,19]. Hypoxia stimulates fibrogenesis and immune cell infiltration in adipose tissue [20,21,22].

Impaired adipose tissue in obesity triggers chronic low-grade inflammation by activating nuclear factor-kappa B (NF-κB) and Janus kinase (JNK)-mediated inflammatory signaling pathways [23,24,25,26,27]. Activation of the NF-kB pathway in hypertrophied adipocytes leads to the increased expression of pro-inflammatory cytokines such as interleukin (IL)-6, tumor necrosis factor-α (TNF-α), interferon-γ (IFN-γ), IL-1β (IL-1β), and C-reactive protein (CRP), which stimulate immune cells and trigger an inflammatory response [25,28,29]. Release of pro-inflammatory cytokines and free fatty acids in the circulation results in insulin resistance in other metabolic tissues such as the liver and skeletal muscle [25,30].

### 3.2. Role of Macrophage Infiltration and Altered Adipokine Signaling

Immune cells within obese adipose tissue play a crucial role in generating inflammatory responses [31,32]. Major remodeling in obese adipose tissue is marked by a rise in infiltration of immune cells such as pro-inflammatory M1 macrophages [33]. Obese adipocytes secrete chemokines such as MCP1 and CXCL12, which recruit M1 macrophages into adipose tissue [34,35,36,37]. These adipose tissue macrophages activate inflammatory signaling pathways and trigger production of pro-inflammatory cytokines such as IL-1β, TNF-α, and IL-6 [38,39]. Pro-inflammatory M1 macrophages promote low-grade inflammation and insulin resistance in obese adipose tissue [26]. In addition to adipocyte hypertrophy and hypoxia, pro-inflammatory cytokines secreted from M1 macrophages cause macrophage polarization from M2 to M1 [40,41]. Adipose tissue macrophage infiltration is mainly confined to visceral adipose tissue, suggesting that visceral adipose tissue is the major adipose depot containing the pro-inflammatory macrophages in obesity [34,42,43]. Adipose tissue macrophages are the major immune cells responsible for the release of pro-inflammatory cytokines in obese adipose tissue [25,44,45]. In obese adipose tissue, these macrophages accumulate around dead and dying adipocytes and form a crown-like structure. Macrophages within this crown-like structure shift to an M1 phenotype and secrete pro-inflammatory cytokines, which recruit more immune cells to adipose tissue [46,47].

Adipose tissue secretes a wide range of adipocyte-specific hormones called adipokines that regulate appetite, metabolic processes, insulin sensitivity and inflammation [48,49,50,51,52]. Numerous adipokines are expressed in adipose tissue, and they can function either locally in a paracrine or autocrine manner or at a distance in an endocrine manner [53]. This enables adipose tissue to communicate with other organs during metabolic disorders. Altered adipokine secretion by adipocytes is linked to chronic inflammation and obesity-related metabolic disorders [50,54]. In this review, the focus is directed toward two main adipokines, adiponectin and leptin.

Adiponectin is mainly secreted from mature adipocytes and regulates energy homeostasis and insulin sensitivity in adipose tissue, skeletal muscle and liver [55]. Adiponectin acts predominantly as a key anti-inflammatory adipokine. It suppresses chronic inflammation by inhibiting the NF-κB signaling pathway and preventing the generation of pro-inflammatory cytokines including IL-6 and TNF-α [56]. Adiponectin activates AMPK via downstream activation of p38 MAPK and PPARα, which improves insulin sensitivity in skeletal muscle [57,58]. Cell culture and murine studies have found that AMPK activation by adiponectin enhances fatty acid oxidation and attenuates lipogenesis, thus reducing hepatic steatosis [59,60].

Adiponectin suppresses the infiltration of adipose tissue macrophages into adipose tissue via inhibition of adipocyte apoptosis [41,61]. Adiponectin induces the proliferation of anti-inflammatory M2 macrophages and suppresses the proliferation of pro-inflammatory M1 macrophages. In addition, adiponectin induces macrophage polarization from M1 to M2 in adipose tissue [62,63,64]. Pro-inflammatory cytokines such as TNF-α and IL-6 suppress the expression of adiponectin in adipocytes in obese rodents and humans [65,66].

Leptin, an adipokine secreted mainly from adipocytes, is a satiety hormone that controls long-term energy balance by reducing appetite [67,68]. In addition to controlling hunger, leptin also serves as a pro-inflammatory adipokine [69,70]. Obese subjects show increased levels of leptin in the circulation, leading to leptin resistance. Leptin resistance occurs due to impaired leptin signaling, leptin transport or the hypothalamic neural circuit that regulates energy homeostasis [71,72]. Leptin triggers pro-inflammatory signaling pathways via JAK2/STAT3, JAK2/PI3K/Akt, and JAK2/ERK1/2 (MAPK) [73]. Leptin-mediated NF-κB signaling also promotes secretion of pro-inflammatory cytokines [70,74]. Hyperleptinemia induced by obesity stimulates the production of pro-inflammatory cytokines such as TNF-α, IL-6, IL-1β, and IFN-γ and inhibits the production of the anti-inflammatory cytokine such as IL-4 [75,76].

### 3.3. Impact of Inflammation on Insulin Resistance, Oxidative Stress, and Endothelial Dysfunction

Human studies and experimental animal models provide evidence that chronic inflammation of adipose tissue plays a substantial role in metabolic dysregulation and contributes to the development of obesity-related insulin resistance, oxidative stress, and endothelial dysfunction [26,77]. The release of pro-inflammatory cytokines such as TNF-α, IL-6, and IL-1β disrupts insulin signaling, generates ROS and promotes vascular damage [25,78,79]. Secretion of pro-inflammatory cytokines from hypertrophied adipocytes disrupts insulin signal transduction, thus affecting glucose and lipid homeostasis and reducing insulin sensitivity, leading to the development of insulin resistance [28,80]. Pro-inflammatory cytokines such as TNF-α, IL-1β and IL-6 released by M1 macrophages activate serine kinases such as JNK and IKKβ. The activated serine kinases phosphorylate insulin receptor substrate-1 (IRS-1) at specific serine residues, leading to proteasomal degradation of IRS-1 and disruption of insulin signaling, which further leads to insulin resistance [81,82,83,84].

Chronic inflammation is one of the possible contributors to oxidative stress in obesity. The release of pro-inflammatory cytokines like TNF-α and IL-6 from obese adipose tissue triggers immune cells, particularly macrophages, to produce ROS, resulting in generating systemic oxidative stress [85,86]. On the other hand, elevated ROS levels are involved in the secretion of pro-inflammatory molecules and expression of adhesion molecules and growth factors via activation of NF-κB and the NADPH oxidase pathway [87,88]. Increased ROS production leads to metabolic disturbances such as insulin resistance, dyslipidemia and endothelial dysfunction [89,90]. Increased ROS levels during oxidative stress activate several Ser/Thr kinases such as JNK, p38 MAPK, and IKKβ. These kinases disrupt insulin signaling via phosphorylating the insulin receptor (IR) and insulin receptor substrates (IRS-1/2) on serine residues, leading to the development of insulin resistance [91,92,93].

The imbalance resulting from overproduction of pro-inflammatory cytokines coupled with diminished release of anti-inflammatory mediators drives systemic inflammation and oxidative stress, ultimately contributing to endothelial dysfunction [78]. Excess release of pro-inflammatory factors such as TNF-α, endothelin-1 and ROS generates an imbalance between vasodilators and vasoconstrictors. Under these conditions, endogenous endothelial NO synthase (eNOS) uncoupling occurs with production of free radicals that directly harm endothelial cells and that also lower vasodilatory nitric oxide bioavailability, bringing about endothelial impairment [94,95,96].

## 4. Vascular Consequences of Obesity

### 4.1. Atherosclerosis and Myocardial Infarction: Inflammatory and Metabolic Drivers of Plaque Formation

Obesity is a primary risk factor for cardiovascular disease and significantly contributes to the development of atherosclerosis and myocardial infarction [97,98]. Atherogenesis is initiated by the dysfunction of endothelial cells, which leads to increased permeability and retention of low-density lipoproteins (LDL) within the arterial intima. Injured endothelium secretes cytokines and adhesion molecules that chemoattract monocytes. These monocytes penetrate the breached wall and differentiate into macrophages in the arterial intima. Retained LDL particles within the intima become oxidized and then engulfed by macrophages, resulting in the formation of foam cells [99,100]. The combination of lipid-laden foam cells and extracellular lipid deposits allows for the formation of the fatty streak, an early atherosclerotic lesion [101]. Obesity accelerates this process by inciting chronic inflammation which, in turn, induces a pro-atherosclerotic phenotype resulting in production of pro-inflammatory adipokines/cytokines, dyslipidemia, insulin resistance, and elevated aldosterone. This elevation in aldosterone is caused by adipocyte-derived mineralocorticoid-releasing factors which link adipose expansion to sodium retention, arterial stiffening and systemic hypertension through the renin–angiotensin–aldosterone system (RAAS) [102]. These drivers promote oxidative stress and endothelial hyperpermeability, leading to an infiltration of lipids into the vascular wall [103,104]. The nucleotide-binding oligomerization domain-like receptor containing pyrin domain 3 (NLRP3) inflammasome, an inflammatory protein that promotes cytokine release and pyroptosis, also plays a role in atherosclerosis. Once activated by cholesterol crystals and oxidized LDL in the vessel wall, this cytoplasmic complex incites vascular inflammation [105].

While systemic lipid dysregulation is strongly associated with plaque formation, recent studies have shifted the focus toward the “outside-in” signaling, mediated by perivascular adipose tissue. In the normal physiological state, perivascular adipose tissue functions by maintaining vascular homeostasis through the promotion of pro-vasodilatory adipokines such as adiponectin, omentin and adipocyte-derived relaxing factor (ADRF) [106]. However, in the setting of obesity and chronic metabolic stress, perivascular adipose tissue undergoes pathological remodeling characterized by a pro-inflammatory state and loss of its vasoprotective properties. This dysfunctional perivascular adipose tissue releases cytokines such as interleukin-6 (IL-6), IL-17, leptin, resistin, and tumor necrosis factor-α (TNF-α) into the adjacent arterial adventitia, while also reducing production of beneficial adipokines and nitric oxide [107,108].

Along with adipose-driven inflammation, obesity is also associated with changes in the gut manifested as metabolic endotoxemia, an upstream factor in the development of vascular injury [109]. Chronic obesity and high-fat diets disrupt the intestinal mucosal barrier, resulting in increased permeability of lipopolysaccharide (LPS) into the systemic circulation [110]. Circulating LPS acts as a potent ligand for toll-like receptor 4 (TLR4) expressed on endothelial cells and circulating monocytes. This process triggers the MyD88-dependent signaling pathway and activates the NF-kB pathway [111]. This signaling cascade induces endothelial expression of adhesion molecules, including intercellular adhesion molecule-1 (ICAM-1), vascular cell adhesion molecule-1 (VCAM-1), and E-selectin, while also enhancing secretion of pro-atherogenic chemokines such as monocyte chemoattractant protein-1 (MCP-1) [112]. A key consequence of activation of this cascade is increased leukocyte adhesion and transmigration, which triggers and amplifies vascular inflammation, contributing to early atherosclerotic changes and plaque development [113].

### 4.2. Cerebrovascular Disease and Stroke

The impact of obesity on cerebrovascular health begins early in life, showing a correlation with changes in vascular development and the integrity of blood vessels [114]. Elevated BMI during adolescence and young adulthood is robustly associated with a heightened risk of cerebrovascular disease, including early-onset ischemic and hemorrhagic stroke [115,116]. The link between obesity and elevated stroke risk is driven by comorbidities such as hypercholesterolemia, hypertension, and diabetes mellitus [117,118,119]. Mechanisms of obesity-induced cerebrovascular endothelial dysfunction include insulin resistance, inflammation from reactive oxygen species, altered adipokine secretion, and blood pressure elevation [120,121].

Obesity drives adaptive vascular remodeling as a response to maintaining wall tension and minimizing wall stress to allow for adequate blood flow [122]. Chronic obesity leads to structural alterations in the cerebral vasculature and has been shown to promote inward eutrophic remodeling of the cerebral resistance arteries. This leads to a decrease in the lumen-to-wall ratio and raises cerebrovascular resistance [123]. Using an experimental model of obesity and stroke in Sprague–Dawley rats, Deutsch et al. showed that obese rats that had been fed a high-fat diet exhibited increased post-ischemic brain damage following middle cerebral artery occlusion compared to rats fed a control diet [124]. Compared to non-obese rats, the obese rats also exhibited increased vascular stiffness with increased expression of collagen type 1 and MMP-2 in the cerebral vessels. This loss of elasticity and increased stiffness contributed to more severe brain injury after a stroke by reducing the vessels’ ability to handle pressure and protect the blood–brain barrier. Osmond et al. used the Zucker rat genetic model of obesity without diabetes, which has a mutation in the leptin receptor, to show an increase in inward remodeling of the middle cerebral artery and more severe infarction following ischemic brain injury in obese compared to lean Zucker rats [125]. Additionally, experimental studies with humans and the use of three-dimension high resolution magnetic resonance vessel wall imaging (3D HRMR-VWI) have demonstrated that obesity induces segment-specific remodeling in cerebral arteries. A single-center cross-sectional study from China enrolled 96 persons between the ages of 18 and 50 years and grouped them by BMI into normal weight, overweight and obese categories. Participants underwent 3D HRMR-VWI of the internal carotid artery and middle cerebral artery. The study found compensatory dilation of the right internal carotid artery C3 segment in overweight individuals compared to those of normal weight, but luminal retraction, interpreted as failure of compensatory mechanisms, in the obese group [126]. In the obese group, the left C1, C2, and C6 segments were significantly thickened. Perez-Corredor et al. used male Wistar albino rats to examine how obesity affects microvascular endothelial cells and astrocytes in vitro. Obesity and dyslipidemia were induced in the rats with fructose consumed in their drinking water, while control rats were given pure water. When cultured microvascular endothelial cells were incubated in plasma from the obese and lean rats, plasma from the obese rats caused endothelial damage and increased extracellular vesicle (EV) release. The EVs released by the cultured endothelium were isolated and added to astrocytes in culture, and astrocytes exposed to EVs originating from endothelial cells exposed to obese plasma exhibited increased NF-kB signaling. When the rats were subjected to vascular occlusion and cerebral ischemia, post-stroke mortality was significantly higher in the obese animals with more severe motor consequences and augmented glial activity [127]. Collectively, these findings show that obesity remodels cerebral arteries, impairs vascular function and increases susceptibility to ischemic injury.

Obesity serves as a substantial contributor to cerebral small-vessel disease, likely by inducing chronic low-grade inflammation and metabolic dysfunction within the microvascular environment [123,128]. Systemic insulin resistance, SIRT1 downregulation and hypertension may impair nitric oxide bioavailability, triggering reduced microvascular blood vessel density and tissue perfusion, leading to decreased neurovascular coupling [129,130,131]. These changes promote structural remodeling of small vessels, including segmental luminal narrowing and increased vascular stiffness. As a result, vascular resistance increases, reducing the brain’s capacity to effectively recruit collateral circulation during ischemic stress [132,133].

Obesity driven inflammation significantly compromises the blood–brain barrier [134]. This process is mediated by a pro-inflammatory adipokine profile that activates matrix metalloproteinases such as MMP-9 within the cerebral microvasculature [135]. Elevated levels of MMP-9 lead to the degradation of essential tight junction proteins such as claudin-5 and occludin, which maintain the restrictive permeability of the blood–brain barrier [134,136]. This allows for an increase in plasma proteins and circulating immune cells to extravasate into the brain parenchyma, promoting neuroinflammation, cerebral edema, and hemorrhagic transformation due to MMP-mediated degradation [137]. The disruption in the blood–brain barrier and elevated MMP-9 levels resulted in more severe ischemic strokes, larger infarct volumes, and worse neurological outcomes [138].

## 5. Metabolic Derangements: Dyslipidemia, Hypertension, and Diabetes

### 5.1. Metabolism in Obesity

Obesity is closely associated with metabolic abnormalities that increase the risk of cardiovascular disease and may also contribute to cognitive decline [139,140]. Dyslipidemia, hypertension, and type 2 diabetes frequently co-occur in persons with excess adiposity. These conditions are exacerbated by adipose tissue dysfunction, chronic inflammation, insulin resistance, and endothelial injury [103,141]. In humans, hypertrophic adipocyte expansion and visceral adiposity are associated with insulin resistance [142]. These processes contribute to atherosclerosis, myocardial dysfunction, cerebrovascular disease, and impaired brain perfusion [143,144]. Thus, metabolic complications of obesity are important pathways through which systemic inflammation can affect both cardiovascular and cognitive health [4].

### 5.2. Dyslipidemia in Obesity and Its Contribution to Vascular Inflammation

Dyslipidemia is a common metabolic consequence of obesity and is a major contributor to atherosclerotic cardiovascular disease. The typical atherogenic lipid pattern seen in obesity includes elevated triglycerides, increased free fatty acids, lower HDL, dysfunctional HDL particles, and a greater proportion of small dense LDL particles [141,145]. These abnormalities are strongly influenced by insulin resistance and adipose tissue dysfunction, which both drive increased hepatic VLDL production and impair clearance of triglyceride-rich lipoproteins [146].

The vascular effects of dyslipidemia are closely tied to inflammation. LDL particles retained in the arterial wall can become oxidized and trigger recruitment of monocytes and macrophages, leading to foam cell formation and plaque development [143]. This process is amplified by obesity-associated inflammation. Adipose tissue releases cytokines and adipokines that promote oxidative stress, endothelial dysfunction, and vascular remodeling [103]. The combination of abnormal lipid handling and chronic inflammation creates an environment that favors atherosclerotic plaque formation and progression.

Dyslipidemia may also contribute to brain injury through vascular and nonvascular pathways. Atherosclerosis and small-vessel disease reduce cerebral perfusion and increase risk for ischemic injury [147]. The NLRP3 inflammasome is activated after ischemic stroke, including in circulating human leukocytes, and experimental models of cerebral ischemia show that NLRP3 activation promotes neuroinflammation and more severe brain injury [148,149,150].

Recent clinicopathologic evidence suggests that carotid artery disease may contribute to cerebral small-vessel disease and dementia rather than serving only as a source of large-vessel stroke. Kitajima et al. examined 159 postmortem internal carotid artery samples with corresponding brain tissue from persons with stroke or dementia and evidence of cerebral small-vessel disease [151]. Greater carotid stenosis and sclerosis were associated with clinical stroke, brain infarction, higher small-vessel disease pathology scores, and a greater total burden of vascular lesions. Carotid stenosis was also associated with dementia caused by cerebrovascular disease and with mixed Alzheimer’s disease and small-vessel disease pathology, particularly subcortical and white-matter lesions in the anterior circulation. These findings support a role for carotid atherosclerosis and altered cerebral perfusion in linking systemic vascular disease to cerebral small-vessel injury and cognitive decline.

Altered lipid signaling may also affect neuronal membrane composition, synaptic function, and neuroinflammatory responses [144,152,153]. The relationship between peripheral lipid levels and cognitive decline is complex and may vary with age, but lipid dysregulation remains a plausible pathway linking obesity to vascular cognitive impairment and neurodegeneration [152,154].

### 5.3. Epicardial Adipose Tissue as a Local Inflammatory Source Affecting the Myocardium

Epicardial adipose tissue is a visceral fat depot located between the myocardium and visceral pericardium. It is different from subcutaneous fat because it lies directly against the heart and coronary arteries without a fascial barrier, allowing local paracrine and vasocrine signaling [155]. Under normal conditions, epicardial adipose tissue may serve as an energy reservoir for the myocardium and may secrete protective adipokines. In obesity and metabolic disease, this tissue expands and becomes more inflammatory and lipotoxic.

Expansion of epicardial adipose tissue is associated with macrophage infiltration, oxidative stress, adipokine imbalance, and increased production of inflammatory mediators [156]. Proximity to the myocardium and coronary arteries allows excess epicardial fat to directly affect coronary atherosclerosis, myocardial fibrosis, diastolic dysfunction, and arrhythmogenesis [157,158]. This local inflammatory activity may help explain why visceral and ectopic fat distribution can predict cardiovascular risk more accurately than body mass index alone.

Epicardial fat may injure the heart through several mechanisms, including inflammation, accumulation of toxic lipid intermediates, mitochondrial dysfunction, oxidative stress, and impaired myocardial metabolism. These effects are relevant to coronary artery disease, atrial fibrillation, heart failure with preserved ejection fraction, and other obesity-related cardiac disorders [157,159]. Epicardial adipose tissue therefore represents a local inflammatory connection between systemic obesity and myocardial injury.

### 5.4. Obesity-Related Hypertension and Endothelial Dysfunction

Hypertension is one of the most common cardiovascular complications of obesity and, when these two conditions co-occur, they hasten damage to the heart and vasculature, leading to increased risk of myocardial infarction, heart failure, stroke, and vascular cognitive impairment [160]. Excess adiposity contributes to hypertension through several mechanisms, including sympathetic nervous system activation, stimulation of the renin–angiotensin–aldosterone system, renal sodium retention, physical compression of the kidneys by visceral and perirenal fat, insulin resistance, and chronic inflammation [161]. These pathways increase vascular resistance and intravascular volume while also promoting target organ injury.

Endothelial dysfunction is central to obesity-related hypertension [162]. The endothelium normally regulates vascular tone, leukocyte adhesion, thrombosis, and barrier function. In obesity, inflammatory cytokines, reactive oxygen species, dyslipidemia, and insulin resistance reduce nitric oxide bioavailability and impair endothelium-dependent vasodilation [163]. Loss of endothelial homeostasis favors vasoconstriction, vascular stiffness, thrombosis, and continued inflammatory cell recruitment [164].

The cerebral circulation is particularly vulnerable to long-standing hypertension and endothelial injury [165]. Hypertension contributes to cerebral small-vessel disease, white-matter damage, microinfarcts, blood–brain barrier dysfunction, and impaired neurovascular coupling [144,166]. These changes can reduce cognitive reserve and increase susceptibility to vascular cognitive impairment and Alzheimer-type pathology. Obesity-related hypertension therefore links metabolic disease to heart and brain dysfunction through shared vascular pathways.

### 5.5. Type 2 Diabetes as a Major Amplifier of Vascular Risk and Cognitive Decline

Type 2 diabetes is one of the most important metabolic complications of obesity and greatly amplifies cardiovascular and cognitive risk. Insulin resistance, hyperglycemia, dyslipidemia, oxidative stress, and chronic inflammation contribute to endothelial dysfunction, vascular stiffness, and accelerated atherosclerosis [167,168]. Advanced glycation end products and inflammatory mediators further damage the vascular wall and promote microvascular and macrovascular disease [169]. As a result, persons with type 2 diabetes have increased risk of coronary artery disease, heart failure, peripheral artery disease, and ischemic stroke.

The relationship between diabetes and cognitive decline is supported by substantial epidemiologic evidence [170,171]. A meta-analysis of prospective studies found that diabetes was associated with increased risk of cognitive impairment and dementia [172]. Earlier meta-analytic work also reported increased risk of all-type dementia, Alzheimer’s disease, and vascular dementia among individuals with diabetes [173]. Although vascular injury is a major contributor, diabetes may also affect cognition through direct effects on the brain.

Evidence suggests that brain dysfunction may begin early in the course of obesity and type 2 diabetes [174]. In a small resting-state functional magnetic resonance imaging study, Yu et al. described abnormal brain network organization in newly diagnosed overweight or obese persons with type 2 diabetes compared with controls [175]. The network abnormality was characterized by reduced small-world organization and global efficiency, together with increased characteristic path length. These findings indicate less efficient communication across functional brain networks even near the onset of diabetes. Chen et al. found that a higher C-reactive protein–triglyceride–glucose index, which combines measures of systemic inflammation and insulin resistance, was associated with mild cognitive impairment and predicted incident cognitive impairment in clinical and nationwide cohorts [176]. The association was also observed during normal glucose regulation and prediabetes, suggesting that cognitive risk may emerge before longstanding or advanced diabetes is established.

Insulin signaling is important for neuronal survival, synaptic plasticity, mitochondrial function, and brain energy metabolism. Brain insulin resistance has been implicated in neuroinflammation, impaired amyloid-β and tau metabolism, oxidative injury, and synaptic dysfunction, although the relationship between diabetes and Alzheimer pathology remains complex [177]. Experimental evidence also indicates that high glucose may directly increase neuronal vulnerability. Lima et al. exposed differentiated SH-SY5Y neuron-like cells to high glucose and observed concentration- and time-dependent reductions in cell viability, together with increased mitochondrial superoxide production, oxidative DNA damage, and apoptosis [178]. High-glucose exposure also intensified the injury caused by hydrogen peroxide and okadaic acid, suggesting that hyperglycemia may amplify neuronal cell susceptibility to additional oxidative and neurotoxic insults. Although these in vitro findings cannot be directly extrapolated to patients, they provide a plausible cellular mechanism by which chronic hyperglycemia may contribute to neuronal dysfunction and neurodegeneration. Thus, type 2 diabetes may contribute to cognitive dysfunction through both vascular and neurodegenerative mechanisms.

Taken together, dyslipidemia, epicardial adipose tissue expansion, hypertension, and type 2 diabetes are interrelated metabolic consequences of obesity. These conditions converge on inflammation, endothelial dysfunction, and vascular injury. They increase risk for cardiovascular disease and may also compromise cerebral perfusion and cognitive health [144]. Addressing these metabolic abnormalities is therefore central to preventing cardiovascular and cognitive complications of obesity.

## 6. Obesity and Cognitive Function

### 6.1. Obesity and Brain Structure

Obesity is associated with structural changes in the brain that can impair cognitive function. These changes are seen on imaging as lower brain volume and increased white-matter hyperintensities, both considered indicators of accelerated brain aging [179,180]. These relationships among obesity, brain structure and cognition are modulated by factors such as age, fat distribution, duration of obesity, and the presence of other metabolic disorders [180]. Increased visceral and ectopic fat have been associated with lower cognitive scores and reductions in brain volume [181]. The mechanisms linking obesity to cognitive impairment include abnormal lipid metabolism, insulin resistance, vascular injury, oxidative stress, and neuroinflammation [182,183]. These processes may affect neuronal function directly and may also increase susceptibility to cerebrovascular disease and neurodegeneration.

### 6.2. Lipid Metabolism in the Brain: Transport, Storage, and Homeostasis

Lipids are essential for normal brain structure and function. Cholesterol, phospholipids, fatty acids, and other lipids are required for neuronal membranes, myelin formation, synaptic activity, and intracellular signaling. The brain maintains a largely independent system of lipid metabolism because most cholesterol-containing lipoproteins in the circulation do not readily cross an intact blood–brain barrier [184,185]. Thus, most cholesterol required by the central nervous system must be produced and regulated within the brain [186].

Astrocytes play a major role in maintaining brain cholesterol levels. In the adult brain, astrocytes synthesize cholesterol and package it into apolipoprotein E (ApoE) particles. ApoE, the most abundant CNS lipoprotein, transports cholesterol to neurons, where it is used for membrane maintenance, synapse formation, and cellular repair [185,187]. Neurons, in turn, rid themselves of toxic fatty acids by transferring them to astrocytes via ApoE for sequestration and metabolism [188]. Astrocytes take up fatty acids released by neurons and store them temporarily in lipid droplets, protecting neurons from lipotoxicity, oxidative stress and cellular injury. Excess neuronal cholesterol can also be converted to 24S-hydroxycholesterol, which is able to cross the blood–brain barrier and enter the circulation for elimination [189,190]. Oligodendrocytes also exhibit high cholesterol utilization in the formation and maintenance of the rich lipid membranes of the myelin sheath [191]. Oligodendrocytes acquire cholesterol through de novo synthesis and can also receive it from astrocytes [192]. Lipid production, transport, storage, and removal must be balanced in order to support normal neuronal and cognitive function.

### 6.3. Dyslipidemia and Neurodegeneration: Altered Lipid Signaling and Membrane Composition

Disruption of lipid homeostasis has been linked to Alzheimer’s disease and other neurodegenerative disorders. Cholesterol, phospholipids, sphingolipids, and fatty acids influence the structure and fluidity of neuronal membranes. Changes in the amount or distribution of these lipids can interfere with receptor activity, synaptic transmission, and communication between brain cells [193].

Membrane lipids also affect the processing of proteins involved in Alzheimer’s disease. Changes in cholesterol and sphingolipid composition alter biophysical characteristics of neuronal cell membranes and thus may influence amyloid precursor protein processing, amyloid-β production and clearance, tau phosphorylation, and inflammatory signaling [194,195]. Abnormal lipid metabolism enhances lipid peroxidation and production of ceramides, and this has been postulated to foster a pro-amyloidogenic milieu in the brain [196]. Amyloid-β can also disrupt the lipid bilayer [195,197].

Experimental findings indicate that lipids can directly affect tau aggregation and toxicity [198,199]. Ali et al. found that the fatty acid composition of phosphatidylserine altered aggregation of the largest tau variant, the 2N4R tau isoform, while the 1N4R isoform, which differs primarily in N-terminal composition from 2N4R, was less affected [200]. Phosphatidylserine also changed the toxicity of both tau isoforms, and fibrils formed in its presence produced greater mitochondrial injury after cellular uptake. Molecular dynamics simulations from Chowdhury et al. similarly showed that tau fibrils interact differently with charged and uncharged lipid membranes and that these interactions can alter β-sheet structure and disrupt the lipid bilayer [201]. These findings suggest that membrane lipid composition can influence the formation, structure, and neurotoxicity of tau fibrils.

Altered lipid metabolism has been identified in persons with Alzheimer’s disease, although it remains uncertain whether these abnormalities initiate neurodegeneration or are secondary, developing in response to it. Abnormal lipid accrual may also contribute to cellular injury [202]. Lipid droplets normally protect cells by storing fatty acids that could otherwise become toxic. Persistent lipid accumulation, however, may impair mitochondrial function and increase oxidative stress and inflammation. Accumulation of lipid droplets in astrocytes and microglia has been observed with aging and in neurodegenerative disease [203]. This may reduce the ability of glial cells to support neurons and clear damaged cellular material.

It is important to distinguish peripheral dyslipidemia from lipid abnormalities within the brain. Circulating cholesterol does not directly reflect brain cholesterol because the two systems are largely separated by the blood–brain barrier, as shown in numerous human and murine studies [204,205,206]. Dyslipidemia may nevertheless affect cognition by promoting atherosclerosis, cerebral small-vessel disease, endothelial dysfunction, and blood–brain barrier injury [183,184]. The evidence linking dyslipidemia to blood–brain barrier dysfunction is derived from both experimental animal models and human studies, but direct causality has not been established [207].

A systematic review and meta-analysis by Wee et al. evaluated 17 longitudinal cohort studies examining midlife lipid levels and later cognitive outcomes [208]. The strongest evidence involved elevated total cholesterol during midlife, which was associated with a 14% higher risk of incident all-cause dementia. Associations involving LDL, HDL, triglycerides, and cognitive impairment were less consistent across studies. These results suggest that the timing of lipid exposure is important and that dyslipidemia during midlife may have consequences that become apparent many years later. Thus, dyslipidemia may contribute to cognitive decline through both vascular injury and interference with lipid homeostasis within the brain [209].

### 6.4. ApoE, Neurons, and Astrocytes: Molecular Links to Alzheimer’s Disease Risk

ApoE is the principal lipid-transport protein in the central nervous system. It is produced mainly by astrocytes and transports cholesterol and other lipids to neurons for membrane repair, synaptic maintenance, and normal cellular function [210,211]. Microglia and neurons may also produce ApoE, particularly during cellular stress or injury.

Three common ApoE alleles produce the ApoE2, ApoE3, and ApoE4 isoforms of the protein. ApoE4 is the strongest common genetic risk factor for late-onset Alzheimer’s disease, while ApoE2 is generally associated with lower risk, and ApoE3, the most common variant, is neutral with regard to AD risk [212,213].

Lipid abnormalities may also interfere with amyloid-β clearance within the brain and in the peripheral circulation. Cell system and animal experiments have shown that low-density lipoprotein receptor-related protein 1 is an important mediator of amyloid-β transport across the blood–brain barrier and uptake by peripheral organs, particularly the liver [214,215,216]. HDL particles containing ApoE or ApoJ can bind amyloid-β and support its transport, while LDL may facilitate receptor-mediated efflux from the brain. Dyslipidemia or impaired lipoprotein receptor function may disrupt these clearance pathways and promote amyloid-β accumulation, although evidence that lipid-modifying treatment improves amyloid clearance in humans remains limited.

ApoE4 affects more than amyloid-β accumulation. It also alters lipid transport, synaptic function, inflammatory responses, cellular metabolism, and vascular integrity. ApoE4 may interfere with the normal exchange of lipids between neurons and astrocytes. Neurons release excess fatty acids that are taken up and stored by astrocytes. Experimental studies have shown that ApoE4 impairs this process, allowing fatty acids to accumulate within neurons and increasing oxidative and metabolic stress [217]. This disruption may make neurons more vulnerable to injury over time.

Astrocyte-derived ApoE4 may also impair the blood–brain barrier. Experimental studies have associated ApoE4 with loss of endothelial tight junctions, reduced astrocyte support of cerebral blood vessels, and increased blood–brain barrier permeability [218,219]. Blood–brain barrier dysfunction may expose the brain to circulating inflammatory mediators and further contribute to neuronal injury.

The effects of ApoE4 on lipid metabolism may connect genetic risk with several features of Alzheimer’s disease. Abnormal lipid handling can impair neuronal metabolism, increase glial inflammation, weaken the blood–brain barrier, and interfere with amyloid-β and tau regulation [210,212]. Thus, the relationship among ApoE, astrocytes, and neurons provides an important molecular link between lipid metabolism and Alzheimer’s disease risk.

### 6.5. Obesity, Glucose Metabolism, and the Brain: Insulin Resistance, Neuroinflammation, and Cognitive Impairment

Insulin has important functions in the brain in addition to its role in peripheral glucose metabolism. Insulin receptors are present in the hippocampus, cerebral cortex, hypothalamus, and other regions involved in memory, learning, appetite, and energy balance. Brain insulin signaling supports neuronal survival, synaptic plasticity, mitochondrial function, and neurotransmitter activity [220,221].

Recent experimental studies suggest that amyloid-β can impair insulin signaling in cerebral endothelial cells and reduce glucose entry into the brain. Wang et al. found that soluble amyloid-β40 and amyloid-β42 reduced fluorodeoxyglucose uptake and influx in experimental models and decreased expression of GLUT1, the primary glucose transporter at the blood–brain barrier. These effects were associated with reduced AKT phosphorylation and increased thioredoxin-interacting protein expression [222]. Nelson et al. similarly found that acute amyloid-β40 exposure impaired insulin-responsive Akt signaling in human blood–brain barrier endothelial cells [223]. Together, these findings provide a possible link among amyloid accumulation, endothelial insulin resistance, and reduced cerebral glucose availability, although the evidence is derived from cellular and animal models.

Obesity can impair the response of the brain to insulin. Chronic hyperinsulinemia, elevated free fatty acids, oxidative stress, and inflammation may disrupt insulin signaling and reduce transport of insulin across the blood–brain barrier [220]. Brain insulin resistance has been associated with poorer memory, impaired executive function, and abnormal regulation of appetite and body weight.

Impaired insulin signaling may also contribute to Alzheimer-type pathology. Insulin resistance can increase oxidative stress, impair mitochondrial function, interfere with synaptic plasticity, and alter the metabolism of amyloid-β and tau [177,183]. These effects provide a possible explanation for the increased risk of cognitive decline seen in persons with obesity, insulin resistance, and type 2 diabetes [224].

Long-term abnormalities in glucose metabolism may also affect brain structure and cognitive function. Longitudinal studies have found that poor glycemic control and insulin resistance are associated with progressive brain atrophy and poorer cognitive performance [225,226]. These findings support the importance of early identification and management of metabolic dysfunction.

Neuroinflammation is another important pathway linking obesity to cognitive impairment [227,228]. Adipose tissue releases pro-inflammatory cytokines that enter the circulation and may impair endothelial and blood–brain barrier function. These inflammatory signals can activate microglia and astrocytes and contribute to oxidative stress, mitochondrial dysfunction, and synaptic injury [182,229]. Persistent neuroinflammation may interfere with learning and memory and increase vulnerability to age-related cognitive decline.

Taken together, altered lipid metabolism, ApoE-associated dysfunction, insulin resistance, and neuroinflammation provide several pathways through which obesity may affect cognitive health. These mechanisms interact with vascular disease and can impair neuronal metabolism, synaptic function, blood–brain barrier integrity, and cerebral perfusion [182,230]. Maintaining metabolic health may therefore be important for preserving cognitive function and reducing long-term risk for neurodegenerative disease [231].

## 7. Heart–Brain Axis: Shared Mechanisms and Interdependence

### 7.1. Interconnection Between Heart and Brain

The heart and brain share a vasculature, an inflammatory environment, and a dependence on continuous perfusion, but they differ in their capacity to recover from injury. Cardiac tissue retains regenerative reserves, yet the adult brain, particularly in regions most vulnerable to ischemic and metabolic insult, possesses far narrower neurogenic capacity [232]. As a result, the vascular damage from obesity, dyslipidemia, hypertension, and chronic inflammation accumulates differently in the heart and the brain. The heart, at least to some degree, may adapt, remodel, or even partially recover with intervention [233]. The brain typically bears the long-term consequences, appearing decades later as small-vessel disease, cognitive decline, or frank dementia [234]. Increasingly, contemporary evidence reflects this asymmetry, framing the heart and brain as a single coupled system that ages together but recovers differently.

### 7.2. Bidirectional Influence Between Cardiovascular and Cognitive Health

Cardiovascular disease accelerates cognitive decline, though the strength of this effect depends on the underlying cardiac condition [235]. Heart failure is the cleanest clinical example, and a recent Heart Failure Society of America scientific statement concluded that cognitive impairment in heart failure is common enough to affect medication management, symptom recognition, and decisional capacity [236]. In the Cardiovascular Health Study, newly diagnosed heart failure was followed by accelerated long-term cognitive decline compared with participants without heart failure, even in the absence of clinical stroke at baseline [237]. Acute myocardial infarction appears to mark a step-change in cognitive trajectory as well, with a 2023 analysis showing accelerated global and executive decline in the years following myocardial infarction [238]. Atrial fibrillation also has cognitive impact: a 2022 meta-analysis spanning approximately 2.8 million individuals found atrial fibrillation to be associated with cognitive decline and incident dementia even after accounting for overt stroke [239], and a 2019 large prospective-cohort meta-analysis reported a pooled hazard ratio of 1.34 for incident dementia in atrial fibrillation patients with normal baseline cognition [240]. The original Rotterdam analysis remains historically important for proposing that silent embolic infarction and atrial fibrillation-related cerebral hypoperfusion could contribute to dementia even in the absence of clinical stroke, and specifically for raising the possibility that such damage could either mimic Alzheimer disease clinically or unmask latent Alzheimer’s disease pathology in patients with reduced cerebral reserve [241]. Together, these findings link impaired cardiac-to-brain perfusion to cognitive decline.

The reverse direction, in which primary central nervous system pathology alters cardiovascular physiology, is biologically real but less directly studied. Autonomic dysfunction, orthostatic hypotension, and blunted baroreflex sensitivity are repeatedly described in Alzheimer disease, and a 2022 meta-analysis confirmed increased orthostatic hypotension prevalence in populations with Alzheimer’s disease [242]. Parkinson disease, dementia with Lewy bodies, and atypical parkinsonian syndromes, most prominently multiple system atrophy, demonstrate this pattern in its most concentrated form: α-synuclein pathology in central and peripheral autonomic networks produces orthostatic hypotension, impaired heart rate variability, and progressive cardiovascular dysautonomia, often preceding motor or cognitive manifestations by years [243]. Small-vessel disease pathology co-occurs with autonomic dysfunction across neurodegenerative dementia cohorts [244]. Psychiatric comorbidity provides another bridge: depression is independently associated with adverse cardiovascular events and arrhythmic vulnerability, and the bidirectional depression–arrhythmia relationship, mediated through the autonomic nervous system and interoceptive pathways, has been a focus of recent narrative reviews [245,246]. Takotsubo (stress-induced) cardiomyopathy illustrates the acute extreme of this influence, in which profound emotional or neurologic stress transiently stuns the myocardium in the absence of obstructive coronary disease [247]. Cognitive impairment also closes the loop through poorer self-management: a systematic review found dementia and cognitive impairment consistently predict compromised heart-failure self-management, medication adherence, and outcomes [248,249]. This pathway is behavioral rather than biological, but its clinical impact is real: cognitive dysfunction reduces the effectiveness of any cardiometabolic intervention the patient receives.

### 7.3. Common Mediators: Vascular Inflammation, Endothelial Injury, Oxidative Stress, and Impaired Neurovascular Coupling

The pathophysiological mechanisms linking adipose biology to cardiac dysfunction and brain vulnerability overlap and operate as an interconnected system rather than as independent processes. Among the shared mechanisms, endothelial dysfunction has emerged as an important common substrate. In peripheral vasculature, reduced nitric oxide bioavailability, oxidative stress, and glycocalyx damage impair vasodilatory reserve and promote atherogenesis; in the brain, a potentially analogous scenario is blood–brain barrier dysfunction and failure of the neurovascular unit [232,250]. Chronic cerebral hypoperfusion compromises blood–brain barrier integrity via increased endothelial transcytosis and disrupts the capillary-level coupling between neuronal activity and local blood flow [251]. On the other end, chronic hypertension is a potent driver of similar processes: sustained pressure damages cerebral endothelial cells and pericytes, compounds blood–brain barrier dysfunction and progressively degrades capillary function [252]. The practical implication is a relationship of systemic, and not merely local, vascular disease to white-matter injury, impaired cerebrovascular reactivity, and cognitive decline.

Impaired neurovascular coupling represents another pathway through which cardiovascular dysfunction produces cerebrovascular consequences [253,254]. Aortic stiffening, a hallmark of vascular aging, allows greater pulsatile energy to reach the cerebral microcirculation [255]. A study involving a pulse-wave computational model of the entire circulatory system demonstrates that the protective coupling between the left ventricle, aorta, and cerebral vasculature is disrupted by age-related aortic stiffening and altered left-ventricular contractility, plausibly contributing to vascular-related dementia [256].

Williams et al. examined the relationship between cognitive changes and arterial stiffness in a prospective cohort within the Multi-Ethnic Study of Atherosclerosis (MESA) [257]. Utilizing cognitive testing, B-mode carotid ultrasound and brain MRI, they found that higher systolic blood pressure was linked to abnormalities in imaging and cognitive decline in Alzheimer’s disease and Alzheimer’s disease-related dementia. Structural carotid arterial stiffness arising from arterial wall changes was associated with cognitive decline and imaging abnormalities, while load-dependent stiffness attributed to elevated blood pressure was not correlated with longitudinal cognitive decline.

Obesity, to the extent that it is associated with the aforementioned functions, appears to blunt neurovascular coupling responses in midlife [258]. Oxidative stress and chronic low-grade inflammation, detailed in earlier sections, accelerate both processes simultaneously. These observations suggest that cardiometabolic inflammation acts as a systemic rather than tissue-specific phenomenon, with the brain microvasculature representing a particularly vulnerable target [259].

### 7.4. Clinical and Imaging Evidence of Cardiometabolic-Cognitive Interactions

Imaging studies have provided some of the most tangible clinical correlates of the heart–brain axis [260]. Obesity and suboptimal cardiovascular health are associated with reduced brain volume and altered white-matter microstructure on MRI in large community cohorts [261], and white-matter hyperintensity burden predicts cognitive decline across diagnostic categories in a recent meta-analysis [262]. Cardiac and vascular imaging markers show modest but consistent correlations with cognition [263]. Coronary artery calcium and clinical coronary disease are associated with mild cognitive impairment and dementia [264], and carotid intima-media thickness is elevated in dementia cohorts [265].

Life-course analyses add a temporal dimension. Midlife obesity increases later dementia risk [266,267], but a superficially paradoxical signal has repeatedly emerged in which higher body mass index measured in late life appears associated with reduced dementia incidence. The apparent paradox more likely than not reflects some confounding factors. First, unintentional weight loss commonly begins eight to ten years before a dementia diagnosis, producing a reverse-causation signature [267,268]. Second, BMI fails to distinguish fat from muscle mass in older adults; when central adiposity rather than BMI is used, the association remains harmful even in elders, with a pooled analysis of 5.06 million participants demonstrating higher dementia risk with elevated waist circumference in those over 65 [269], and a 2025 Korean cohort of nearly 1 million women reporting an 83% higher vascular dementia risk at waist circumference at or above 95 cm [270]. Third, a small, randomized trial demonstrated that intentional weight loss in obese elderly individuals with mild cognitive impairment modestly improved cognition rather than harming it, reinforcing that the late-life “protective” signal derives from disease-driven unintentional weight loss, rather than a genuine biological benefit of adiposity [271]. Finally, dementia is associated with loss of smell and taste, which can contribute to diminished appetite [272].

Obesity-related obstructive sleep apnea represents another important clinical dimension of the heart–brain axis [273]. In routine clinical practice, untreated sleep-disordered breathing produces cognitive fogginess, impaired alertness, and reduced functional engagement that are clinically apparent even without formal neuropsychological testing [274]. The adiposity–hypoxemia–daytime-function bridge is an important clinical dimension of the heart–brain axis even when longer-term intervention trials of continuous positive airway pressure have returned mixed endpoints, with detailed therapeutic considerations appearing in Section 7.

Recent interventional studies have provided important evidence regarding the temporal window in which cardiometabolic intervention appears most effective. Intensive blood pressure control in the SPRINT-MIND trial reduced the incidence of mild cognitive impairment, although the primary probable-dementia endpoint was not reached [275]. The multidomain FINGER trial in at-risk older adults showed modest but consistent cognitive benefits from combined diet, exercise, cognitive training, and vascular-risk monitoring [276], and large cohort analyses using the American Heart Association’s Life’s Essential 8 construct demonstrate a dose–response relationship between better cardiovascular health and lower dementia risk [277]. By contrast, late-stage cardiometabolic intervention in established Alzheimer disease has repeatedly failed to deliver cognitive or functional benefit. The phase 3 EVOKE and EVOKE+ trials of oral semaglutide in 3808 amyloid-positive patients with mild cognitive impairment or mild dementia, published in March 2026, reported null results on the primary cognitive endpoint and on all prespecified secondaries, including activities of daily living and quality-of-life proxy measures, despite favorable movement in plasma high-sensitivity CRP and cerebrospinal-fluid tau biomarkers [278]. The phase 2b ELAD trial of liraglutide in mild-to-moderate Alzheimer disease similarly did not improve activities of daily living or global clinical decline [279], and the phase 3 EXERT exercise trial in amnestic mild cognitive impairment showed no between-group differences on functional endpoints [280]. Taken together, these results suggest that cardiometabolic interventions are most effective when initiated during the earlier, modifiable window of disease, before cumulative vascular damage becomes entrenched. By contrast, interventions appear less effective once disease is established, at least on current evidence.

These interventional findings have important implications for interpreting the recent estimate that as many as 45% of global dementia cases may have materially attributable risk from modifiable factors, with cardiometabolic and vascular contributors forming the largest mechanistically coherent cluster [3,281]. Because pure vascular dementia accounts for approximately 15% to 20% of dementia diagnoses, with mixed Alzheimer–vascular pathology adding another 10% to 15% [144], a 45% attributable fraction requires that vascular factors contribute to non-vascular dementias as well, a position supported biologically by the high prevalence of mixed pathology at autopsy and by the vascular contributions to Alzheimer pathophysiology outlined above. However, the realized preventable fraction under current intervention effectiveness, as reflected in the partial SPRINT-MIND result, the null EVOKE outcome, and the mixed continuous positive airway pressure literature, is probably smaller than this population-attributable estimate. The appropriate clinical framing is that vascular-risk reduction is a meaningful, and underexploited, lever for cognitive preservation when initiated early enough in the life course, rather than a curative or late-stage rescue strategy. Table 1 provides a summary of the common obesity-related risk factors and disease mechanisms in heart and brain disorders.

## 8. Therapeutic Pathways: Interventions Benefiting Both Heart and Brain

### 8.1. Pharmacologic

Factors contributing to the risk of both brain and heart disease include dyslipidemia, diabetes, atrial fibrillation, heart failure, high serum markers of inflammation, tobacco use, coronary artery disease, and a pro-thrombotic state [282]. Pharmacological therapies can mitigate many of these shared risk factors, and this is particularly true with regard to the vascular system. Employing medications that impact cardiometabolic and vascular function may bring substantial benefit to both heart and brain and is therefore a very active area of study [283,284]. Drug classes with potential dual cardioprotective and neuroprotective effects generally fall into the categories of antihypertensives, lipid-lowering agents, anti-inflammatory therapies, glucagon-like peptide-1 (GLP-1) receptor agonists, and sodium–glucose cotransporter-2 (SGLT2) inhibitors.

#### 8.1.1. Antihypertensives

Antihypertensive pharmacologic therapy reduces stress on the vasculature, and this is favorable for both heart and brain. Hypertension is known to be associated with cognitive decline, and this effect may be attributed, at least in part, to reduced cerebral blood flow [285]. In addition to stroke and vascular dementia, hypertension is also linked to MCI and AD, but whether stringent management of blood pressure can reduce dementia risk is unresolved [286,287]. Clinical trials have studied the relationship between blood pressure and cognition, and several have shown that tighter control of blood pressure does not bring cognitive harm, which was thought to be possible due to lower cerebral perfusion with lower blood pressure [288]. The Effects of Intensive Systolic Blood Pressure Lowering Treatment in Reducing Risk of Vascular Events (ESPRIT) randomized open-label trial studied 11,255 Asian patients aged 50 years and over and found that, compared to standard blood pressure control to a systolic blood pressure target below 140 mm Hg, intensive blood pressure management to a target below 120 mm Hg did not adversely affect global cognitive function over 3 years [289]. The SPRINT MIND randomized trial found that treating 9361 hypertensive patients over age 50 years intensively to attain a systolic blood pressure below 120 mm Hg compared to a less rigorous standard below 140 mm Hg did not find a significant difference in risk of dementia, but did detect a reduced incidence of MCI and a reduced incidence of combined MCI and dementia [275]. These trials suggest that even though hypertensive therapies are expected to decrease the risk of cognitive decline, this is not a linear correlation [290,291]. A study by Sood et al. analyzed over 3300 older community-dwelling persons without baseline dementia found that those who were taking antihypertensive medication at baseline showed significantly slower cognitive decline over an average follow-up of 8.6 years [292]. In some deceased participants, postmortem neuropathology was available and showed less evidence of AD pathology and fewer tau tangles in those who had been receiving antihypertensive treatment independent of blood pressure levels. There also exists a concern that intensive blood pressure control can increase the risk of orthostatic hypotension, which could lead to cognitive decline [293].

#### 8.1.2. Lipid-Lowering Treatment

Lipid-lowering therapies such as statins, protein convertase subtilisin/kexin type 9 (PCSK9) inhibitors, and ezetimibe are similarly therapeutic drugs for the heart and brain likely via multiple mechanisms that include improved endothelial function, reduced atherothrombosis, and lower ischemic stroke risk.

Despite these mechanistic benefits, lipid-lowering agents such as statins demonstrate conflicting results in reducing cognitive impairment. A meta-analysis of randomized controlled trials by Reddin et al. with over 100,000 participants from 15 studies found that lipid-lowering therapy was not significantly associated with lower risk of cognitive impairment or dementia [294]. Another study analyzing a UK Biobank Cohort found a more nuanced effect of lipid-lowering therapies, with high LDL-C being linked to increased AD risk across all groups while low LDL-C was linked to vascular dementia risk in those not taking cholesterol-lowering medications [295].

Statins are the first-line treatment for hyperlipidemia and prevention of cardiovascular disease [296]. There has been controversy surrounding statin effects on cognition and the possible negative impact of aggressive cholesterol lowering since the brain itself is so rich in cholesterol, but a large body of research refutes this [297]. Several meta-analyses have examined the effect of statin use on cognitive impairment in Alzheimer’s disease (AD) and dementia. In a meta-analysis of observational studies with primary outcomes of AD and dementia, Olmastroni et al. found no statistically significant neurocognitive risk associated with statin treatment [298]. In fact, analysis of the pooled data from 36 studies found a statistically significant decrease in the risk of dementia with statins. Additionally, the risks for dementia and AD observed were similar for both lipophilic and hydrophilic statins, suggesting that the impact is not affected by brain penetration and is more systemically driven. A very large meta-analysis of 55 observational studies conducted by Westphal Filho et al. similarly found that statins reduced risk of all-cause dementia, AD and vascular dementia [299]. Further, a subgroup analysis showed that lipophilic statins reduced risk of all-cause dementia while hydrophilic statins did not change all-cause dementia risk. Both hydrophilic and lipophilic statins showed protective effects in AD. The issues of statin potency and lipophilicity continue to be explored [300].

In the Action in Diabetes and Vascular Disease, PreterAx and DiamicroN MR Controlled Evaluation (ADVANCE) study, a randomized, controlled trial focusing on patients aged 55 years and older with type 2 diabetes and known vascular disease or an additional vascular disease risk factor beyond their diabetes, were subjected to either routine or intensive blood pressure lowering and either standard or intensive glucose-lowering targets [301]. In a follow-up of patients from the ADVANCE trial, the relationship between LDL level, statin use and development of dementia or cognitive decline was analyzed. The study found that higher LDL cholesterol was associated with greater odds of dementia or cognitive decline, while statin treatment was not associated with incident dementia risk. A UK Biobank study found that the age of the statin user modulated the cognitive effects of statins [302]. Of the three measures of executive function evaluated in this study (reaction time, working memory, and reasoning), statins produced differential effects on cognitive performance depending on age and the specific aspect of cognitive function tested. This underscores the controversy and complexity in defining the statin effect on cognition and the ways in which modulating factors such as age should be considered.

The profound lipid-lowering therapy with PCSK9 inhibitors such as evolocumab and alirocumab caused concern that cognition would be adversely affected while cardiovascular benefits were clear [303,304]. An open-label extension of a prospective clinical trial of patients taking evolocumab with statin or placebo with statin followed the patients for a median of over 5 years after those who had been on placebo were switched to evolocumab and found no significant decline in cognitive function in patients with LDL levels below 25 mg/dL achieved with two drugs [305]. Similarly, the MEMOGAL study, a smaller-scale prospective real-world observational study from Spain, looked at 158 patients with atherosclerotic cardiovascular disease taking either evolocumab or alirocumab, and found no significant change from baseline in neurocognitive function over 24 months based on MoCA score monitoring with either drug [306].

Data are limited on ezetimibe, a newer lipid-lowering therapy that blocks cholesterol absorption in the intestine. Thus far, it shows a neutral and possibly neuroprotective effect [307,308,309]. The combination of antihypertensive and lipid-lowering therapies may be beneficial for cognitive decline by preventing and minimizing hypoxia, ischemia, hypoperfusion, and blood–brain barrier dysfunction as potential protective mechanisms [310].

#### 8.1.3. GLP-1 Receptor Agonists

GLP-1 receptor agonists are a pharmacologic category being evaluated for effects on the heart and brain. Drugs of this class, which include liraglutide and semaglutide, reduce major adverse cardiovascular events in people with type 2 diabetes and are well-known for their weight loss effects [311,312]. Independent of weight reduction, GLP-1 agonists are thought to have cardioprotective effects by reducing inflammation and postprandial lipemia, while their neuroprotective advantage remains largely hypothetical and related predominantly to preclinical studies showing attenuated neuroinflammation and oxidative stress [313,314,315,316,317,318]. The cardioprotective effects of GLP-1 drugs were established in the SELECT trial, a randomized, double-blind study in which semaglutide or placebo was administered to patients without diabetes with pre-existing cardiovascular disease and overweight/obesity (BMI of 27 or greater) [319]. SELECT showed that weekly semaglutide was superior to placebo in reducing the incidence of death from cardiovascular causes, nonfatal myocardial infarction or nonfatal stroke.

In contrast to their cardiovascular efficacy, neuroprotective effects of GLP-1 treatments remain preliminary, with data coming from fewer and smaller clinical trials. In the Phase 2b Evaluating Liraglutide in Alzheimer’s disease (ELAD) randomized trial, patients with mild to moderate AD without diabetes treated with liraglutide over 12 months showed no change in cerebral metabolic rate of glucose compared to those treated with placebo [279]. However, reduced cognitive deterioration and gray matter loss were detected despite the short study duration. The EVOKE and EVOKE+ phase 3, randomized, placebo-controlled trials evaluated the efficacy and safety of oral semaglutide in persons with amyloid-positive early dementia or MCI and found no slowing of clinical dementia progression with semaglutide (NCT04777396, NCT04777409) [278,320]. Dulaglutide, a GLP-1 receptor agonist, was shown in the Researching Cardiovascular Events With a Weekly Incretin in Diabetes (REWIND) trial to reduce cardiovascular risk in type 2 diabetes, and an exploratory analysis indicated that it may also reduce cognitive impairment [321,322]. A post hoc biomarker analysis found no significant change in AD- related biomarkers over 2 years in aggregate, but neurofilament light levels came down significantly over 2 years in the subset of participants treated with dulaglutide whose initial levels were 56 pg/mL or above [323]. Further, those treated with dulaglutide with a baseline p-tau217 level at or above 0.25 pg/mL exhibited a significant reduction in substantive cognitive impairment when compared to those given the placebo.

Overall, despite their superior cardiovascular efficacy, the cognitive impact of GLP-1 drugs appears to vary depending upon comorbidities such as type 2 diabetes and AD, and much more research is needed to clarify these effects [324].

Finally, SGLT2 inhibitors are useful in the treatment of patients with diabetes, especially with cardiovascular comorbidities [325,326]. The SGLT2 inhibitor drugs empagliflozin and dapagliflozin were studied in clinical trials and shown to reduce the risk of worsening heart failure and cardiovascular death in patients with heart failure [327,328].

SGLT2 inhibitors are lipid-soluble and reach the brain where they may exert both direct and indirect protective effects. Preclinical studies suggest that these medications may preserve blood–brain barrier integrity, reduce pro-inflammatory cytokines, and improve cerebral blood flow [329]. In the rodent brain, empagliflozin induced production of hydrogen sulfide, leading to a lowering of brain oxidative stress [330]. Early translational evidence from the CANVAS trial found that canagliflozin significantly reduced the composite endpoint of cardiovascular death, nonfatal myocardial infarction, or nonfatal stroke in patients with type 2 diabetes and established cardiovascular disease or multiple cardiovascular risk factors [331]. While there are some human studies supporting the ability of these drugs to protect cognitive function in persons with type 2 diabetes, future clinical trials are needed to evaluate the neuroprotective effect of SGLT2 inhibitors given their lipophilic constitution and potentiation of the blood–brain barrier [332,333].

#### 8.1.4. Anti-Inflammatory Treatments

Low-dose colchicine is currently the only FDA-approved drug for reducing low-grade inflammation [334]. It has a variety of mechanisms, including the inhibition of NLRP3 that releases cytokines such as interleukin (IL)-1, IL-1β, and IL-6, as well as inhibiting microtubule polymerization [335]. When paired with aspirin or non-steroidal anti-inflammatory medication, it is a standard first-line treatment for acute pericarditis and for prevention of recurrent pericarditis [336]. In the United States, low-dose colchicine is FDA-approved to reduce the risk of myocardial infarction, stroke, coronary revascularization, and cardiovascular death in adults with established atherosclerotic disease or multiple cardiovascular risk factors [337,338]. A number of randomized trials have shown that adding 0.5 mg of colchicine once daily to guideline-directed medical therapy for secondary prevention of cardiovascular events can help to lower residual ischemic cardiovascular risk [337,339]. In a recent real-world observational study of 614 patients with chronic coronary syndrome, low-dose colchicine use was associated with significant improvements in functional capacity, angina burden, nitroglycerin use, treatment satisfaction, and angina-related quality of life [340].

Evidence of colchicine effects on the brain is less developed and mainly based on preclinical experiments. A recent animal study performed by Liu et al. found that colchicine alleviates ischemic white-matter lesions and cognitive deficits by inhibiting microglial inflammation via the transforming growth factor-beta-activated kinase 1 (TAK1)/MAPK/NF-κB signaling pathway in mice [341]. Furthermore, the COLCOT randomized clinical trial found that low-dose colchicine greatly reduced risk for stroke (HR 0.26 (0.10, 0.70)), suggesting that colchicine may significantly lower ischemic event risk beyond its cardiovascular benefit alone [339]. Taken together, these studies, both preclinical and clinical, show that colchicine, beyond its traditional use in pericarditis, may be beneficial for reducing ischemic risk of stroke in patients with established atherosclerotic cardiovascular disease, chronic coronary disease, or recent myocardial infarction [342].

The anti-IL-1β inhibitor, canakinumab, a fully human monoclonal antibody, was evaluated in the Canakinumab Anti-inflammatory Thrombosis Outcome Study (CANTOS) clinical trial. CANTOS, a randomized, double-blind, placebo-controlled trial, tested the impact of inhibiting IL-1β on the risk of cardiovascular events and found that canakinumab administered every 3 months subcutaneously at a dose of 150 mg significantly lowered the rate of cardiovascular events such as non-fatal myocardial infarction, non-fatal stroke, or cardiovascular death [343]. These results were achieved despite no effect on HDL or LDL cholesterol levels.

IL-6 inhibitors are anti-inflammatory drugs with beneficial implications for the brain and heart. Higher IL-6 levels have been associated with greater risk of cardiovascular death, major adverse cardiovascular events, stroke, and heart failure [344]. The randomized, double-blind phase 2 RESCUE trial showed that IL-6 inhibition with the monoclonal antibody ziltivekimab reduced biomarkers of inflammation and thrombosis pertinent to atherosclerosis [345]. This improvement in thrombotic risk markers and the known increased risk associated with high IL-6 values make these inhibitors a compelling link that may be leveraged to improve mortality associated with cardiovascular and neurovascular diseases. The ongoing randomized phase 3 ZEUS trial (NCT05021835) is currently comparing standard care with the addition of either ziltivekimab or placebo to determine whether ziltivekimab reduces the risk of cardiovascular events such as a myocardial infarction or stroke in patients with cardiovascular disease, chronic kidney disease, and inflammation [346]. This study is estimated to be completed in June 2026 and could provide valuable insight into the hypothesized benefits of ziltivekimab on vascular events.

#### 8.1.5. Anti-Platelet and Anti-Coagulant Medications

Lastly, antiplatelet and anticoagulant therapy have benefits in reducing vascular events and preventing future cardiovascular events, but effects on cognition are unclear [347]. A meta-analysis including 11 randomized clinical trials with 109,860 patients found that antiplatelet therapy was not statistically associated with lower risk of incident cognitive impairment or dementia [348]. The uncertain cognitive benefits of antiplatelet and anticoagulant therapy, despite their well-established cardiovascular benefits in coronary artery disease, leave an important unresolved question [349,350]. Benefits to cognition are most likely in persons with risk of stroke from atrial fibrillation [351,352].

Table 2 presents an overview of the available medications that can be used for cardioprotection and neuroprotection.

### 8.2. Lifestyle Interventions

Lifestyle modifications are a cornerstone preventative measure for cardiovascular health and maintenance of cognitive function. These modifications include, but are not limited to, dietary adjustments, consistent physical activity, and sleep optimization. A nutritious, balanced diet is one of the essential components for optimizing the lipid profile and reducing inflammatory markers in order to protect the vasculature necessary for heart and brain health [353,354]. There is strong evidence that the Mediterranean diet lowers rates of diabetes, improves glycemic control, and reduces incidence of cardiovascular outcomes [355,356]. The traditional Mediterranean diet includes high intake of plant-derived foods and olive oil, moderate consumption of dairy products, poultry and fish, and de-emphasis on red meat and processed foods [357,358]. The Mediterranean diet has been associated with improved composite measures of cognitive function [359].

A cross-sectional study from Italy highlighted the translational significance of the Mediterranean diet by examining the association between adherence to the diet, neuroinflammatory biomarkers, and cognitive status in 92 older participants, 37 diagnosed with mild cognitive impairment (MCI) and 37 without MCI [360]. The study found that stricter adherence to the Mediterranean diet was associated with a lower likelihood of MCI in a dose-dependent manner. MCI was associated with a significantly lower plasma TGF-β1/TNF-α ratio. A study by Pu et al. looked at the relationship between dietary patterns and MCI in patients with diabetes since diabetes is a major risk factor for cognitive impairment [361]. Using the dietary inflammatory index to assess the effect of food components on inflammation, the study found that a higher dietary inflammatory index was associated with MCI in diabetic patients. Additionally, more stringent adherence to the Mediterranean diet was associated with a lower prevalence of MCI. On the other hand, the Western-style dietary pattern consisting of high red meat consumption and low vegetable intake was associated with a higher prevalence of MCI. Based on the literature supporting the benefits of Mediterranean diet adherence in delaying cognitive decline, the Hispanic Community Health Study/Study of Latinos (HCHS/SOL) analyzed brain MRI scans of middle-aged and older participants and found that higher adherence to the Mediterranean diet was associated with better white-matter integrity and preserved microstructure [362].

Another notable diet, the Dietary Approaches to Stop Hypertension (DASH), which emphasizes fruits, vegetables, and low-fat dairy, has been promoted by the American Heart Association to improve cardiovascular health through blood pressure control [363]. Clinical trials found that the DASH diet reduced high-sensitivity cardiac troponin I and hs-CRP over 12 weeks in patients with hypertension, and was associated with lower subjective cognitive decline [363,364]. A hybrid version of the previous two diets, known as the Mediterranean–DASH Intervention for Neurodegenerative Delay, known as the MIND diet, has been associated with a decreased risk of AD, reduced speed of cognitive decline, and fewer neuropathologic changes with AD [365,366,367].

Physical activity is crucial for both neuroprotective and cardioprotective roles. A meta-analysis performed by Iso-Markku et al. analyzing 104 studies with 341,471 participants found that physical activity had a weak association with global cognition, episodic memory, and verbal fluency domains [368]. This is in contrast to other studies and meta-analyses that demonstrate the benefit of aerobic exercise on hippocampal volume, BDNF level, and executive function [369,370]. Similarly, resistance exercise has been shown to improve memory and cognitive function and confer neuroprotective effects [371]. A meta-analysis conducted by Zhang et al. found that concurrent aerobic and resistance training significantly enhances cognitive health, especially in older adults and clinical populations [372]. Overall, despite conflicting reports, physical activity encompassing both aerobic and resistance training is beneficial to cognitive function and improves markers of brain health [373]. This concurs with evidence that sedentary behavior is independently a risk factor for cognitive decline [374,375,376].

A systematic review that looked at types of sedentary behaviors and their association with cognitive outcomes found that time spent on passive activities such as watching television was the most consistently associated with poorer cognitive outcomes [377]. On the other hand, more mentally challenging behaviors such as computer or internet use showed a positive association with better performance in memory and executive functions in 23% of studies.

Finally, sleep optimization is another lifestyle modification with benefits on cardiometabolic and cognitive risk factors [378,379]. Obstructive sleep apnea may damage both heart and brain with periods of oxygen deprivation [380,381]. Research investigating the benefits of continuous positive airway pressure (CPAP) for obstructive sleep apnea found that CPAP treatment did not consistently result in a significantly lower prevalence of cardiovascular events [382,383]. However, cardiovascular risk was lowered with CPAP in the subset of patients who exhibited large drops in blood oxygen or major increases in heart rate during apnea periods [384]. With regard to CPAP and cognition, a study conducted by Velescu et al. found that one year of CPAP adherence improved global cognition associated with obstructive sleep apnea [385]. Similarly, Richards et al. found a statistically significant improvement in psychomotor and cognitive processing speed in patients with obstructive sleep apnea and mild cognitive impairment after one year of CPAP adherence [386]. Feng et al. analyzed data from The China Health and Retirement Longitudinal Study (CHARLS) and reported a U-shaped association between habitual sleep duration and incident cognitive decline in adults aged 45 years and over [387]. Both short sleep duration (less than 6 h) and long sleep duration (exceeding 8 h) were associated with a higher risk of cognitive decline, with modeling placing the optimal baseline at approximately 7.23 h per night. Sleep duration is also important for cardiovascular disease, where sleeping below the 7 h recommended for optimal health poses increased risk for obesity, high blood pressure, and atherosclerosis [388]. Disruption of sleep due to shift work, sleep irregularity, late meal timing, and late chronotype all lead to disruption of circadian rhythm, which has detrimental effects on cardiometabolic health [389].

### 8.3. Integrated Care Models

Integrating these aspects into a multidomain lifestyle has been studied in several clinical trials [390]. The FINGER study randomized a cohort of over 1200 individual patients at risk for cognitive decline into either a multidomain intervention group receiving nutritional guidance, exercise, cognitive training, social activity, and management of metabolic and vascular factors or a control group [276,391]. The intervention group had significantly better cognitive performance at 2-year follow-up compared to the control. This was a landmark study that was generalized to a larger, more diverse US population at risk of dementia with the U.S. Study to Protect Brain Health Through Lifestyle Intervention to Reduce Risk (U.S. POINTER) trial [392]. The US POINTER trial similarly found a benefit to both structured and self-guided interventions, where the structured arm showed greater improvements in global cognition and differed in terms of frequent peer team meetings and a prescribed exercise regimen along with the MIND diet [393]. Thereby, the integration of diet, exercise, cognitive training, and vascular-risk management into a multidomain approach has yielded success in neurocognitive domains.

Cardiovascular conditions have similar models such as the metabolic syndrome care model and the cardiovascular–kidney–metabolic syndrome model, which recognize metabolic syndrome as a progressive pathologic state and encourage interdisciplinary care [394,395,396]. Additionally, the patient-centered medical home (PCMH) model promotes whole-patient, team-based and coordinated care [397]. This model can be similarly applied to dual heart–brain risk, thereby encouraging interdisciplinary care and increasing patient access to resources.

### 8.4. Emerging Approaches

The gut–heart–brain axis is a widely studied framework that focuses on alterations in the intestinal barrier that compromise its integrity and how this can influence cardiac remodeling, atherosclerosis, and cognitive decline [398]. Trimethylamine-N-oxide (TMAO), a metabolite of dietary choline and L-carnitine, acts as a primary mediator in the gut–brain–heart axis. Elevated TMAO levels are linked to atherosclerosis via their vascular inflammatory effects and their pro-thrombotic activation of platelets [399]. In humans, high circulating levels of TMAO are associated with hypertension, increased risk of cardiovascular and thrombotic events and greater platelet reactivity [400,401,402]. Activation of the NLRP3 inflammasome is a key inflammatory mechanism triggered by TMAO [403]. Rat studies have found that TMAO disrupts autonomic nervous system signaling in a way that can turn on a positive feedback loop stimulating the sympathetic nervous system, inciting inflammation and raising blood pressure [404,405,406,407]. TMAO crosses the blood–brain barrier and, based on cell models and animal studies, it has been posited that it may polarize and activate microglia, leading to neuroinflammation [408,409]. Figure 1 illustrates the involvement of TMAO in driving pathological processes in both heart and brain. Although data are mostly preclinical thus far, a meta-analysis of seven studies by Ren et al. found elevated TMAO levels in those with cognitive dysfunction compared to controls [410,411]. This makes TMAO a novel target to be further evaluated for application in the treatment and prevention of neuroinflammation-related cognitive changes.

In murine models, choline TMA lyase inhibitors reduced bacterial TMA formation and improved obesity by rewiring host circadian rhythms [412]. These inhibitors also showed anti-thrombotic activity [413,414].

Another emerging therapy for neurologic and cardiometabolic health is fecal microbiota transplantation [415]. This involves transferring fecal matter from a healthy donor to the gastrointestinal tract of the recipient in an effort to restore a normal gut microbiome in the recipient [416]. It has been used in mouse models of Parkinson’s disease to reduce oxidative stress as well as in cancer treatment [417]. Randomized controlled human trials on fecal microbiota transplantation have shown that the procedure may improve body composition and metabolic health compared with placebo in persons with obesity and metabolic dysfunction [418,419]. A meta-analysis of 10 randomized controlled trials involving 532 patients with metabolic syndrome found that fecal transplantation was associated with higher HDL levels [420]. However, evidence is inconsistent with regard to sustained improvements in body weight, overall body composition, or cardiometabolic health [421].

A focus on nutrition with continuous glucose monitoring and precision nutrition recommendations based on genotype and microbiome are additional new personalized approaches. Continuous glucose monitoring significantly improves HbA1c, fasting glucose, and body weight in patients with type 2 diabetes [422]. Precision nutrition is an avenue through which dietary advice is designed by providing personalized and unbiased nutritional counseling [423]. It encompasses genetics, dietary habits, physical activity, and microbiota to make nutritional recommendations that are beneficial for many metabolic conditions, which serve as risk factors for a variety of heart and brain conditions [424,425]. Genotype studies have found that mutations leading to poor expression or function of the melanocortin 4 receptor gene, which is expressed in the brain and serves as a critical regulator of appetite and body weight, can lead to preferences for specific macronutrients such as fat or carbohydrates [426,427,428]. The cost-effectiveness of precision nutrition interventions remains a challenge [429].

Personalized metabolic therapy involves tailoring medication regimens to the individual’s genetic profile. For example, genetic polymorphisms in the *SLCO1B1* gene result in altered transport of statins and their metabolites into the liver [430]. These polymorphisms can lead to changes in catabolism or activity of statins, affecting their plasma levels, effectiveness in lipid-lowering and likelihood of side effects such as muscle weakness [431]. Metabolomic phenotyping is another precision-medicine advance that identifies biomarkers that can phenotype and categorize the level of glycemic response. This can aid the clinician in determining whether a patient with diabetes will respond better to SGLT2 inhibitors or GLP-1 agonists [432]. Overall, these emerging approaches use specific and personalized technologies to optimize medication delivery, taking into consideration the gut–brain–heart axis [433].

## 9. Conclusions

Obesity threatens heart and brain health through overlapping inflammatory, metabolic, vascular, and neurobiological pathways. The vascular consequences of obesity are particularly important. Obesity promotes atherosclerosis through dyslipidemia, oxidative stress, endothelial activation, macrophage recruitment, and plaque formation. Excess adiposity affects the brain not only through metabolic and inflammatory pathways in the micro- and macrovasculature but also via negative effects on brain volume and structure. Obesity can coexist with both overnutrition and poor nutritional quality, making it necessary to distinguish weight reduction alone from broader improvement in metabolic and vascular health. Stratagems to address and avoid the consequences of obesity should therefore focus not only on weight reduction, but also on preservation of vascular integrity, metabolic health, sleep quality, and cognitive function. The treatment landscape is evolving. Lifestyle interventions remain foundational because physical activity, dietary quality, sleep regularity, and weight management can improve cardiometabolic health and may support cognitive resilience. Prevention and management of the complex pathological effects of obesity on the heart and brain call for a multidimensional, multiprofessional approach that incorporates precision-medicine strategies offering the potential to tailor the treatment plan to an individual’s clinical phenotype, cardiometabolic risk profile, comorbidities, and biological characteristics.

## Figures and Tables

**Figure 1:**
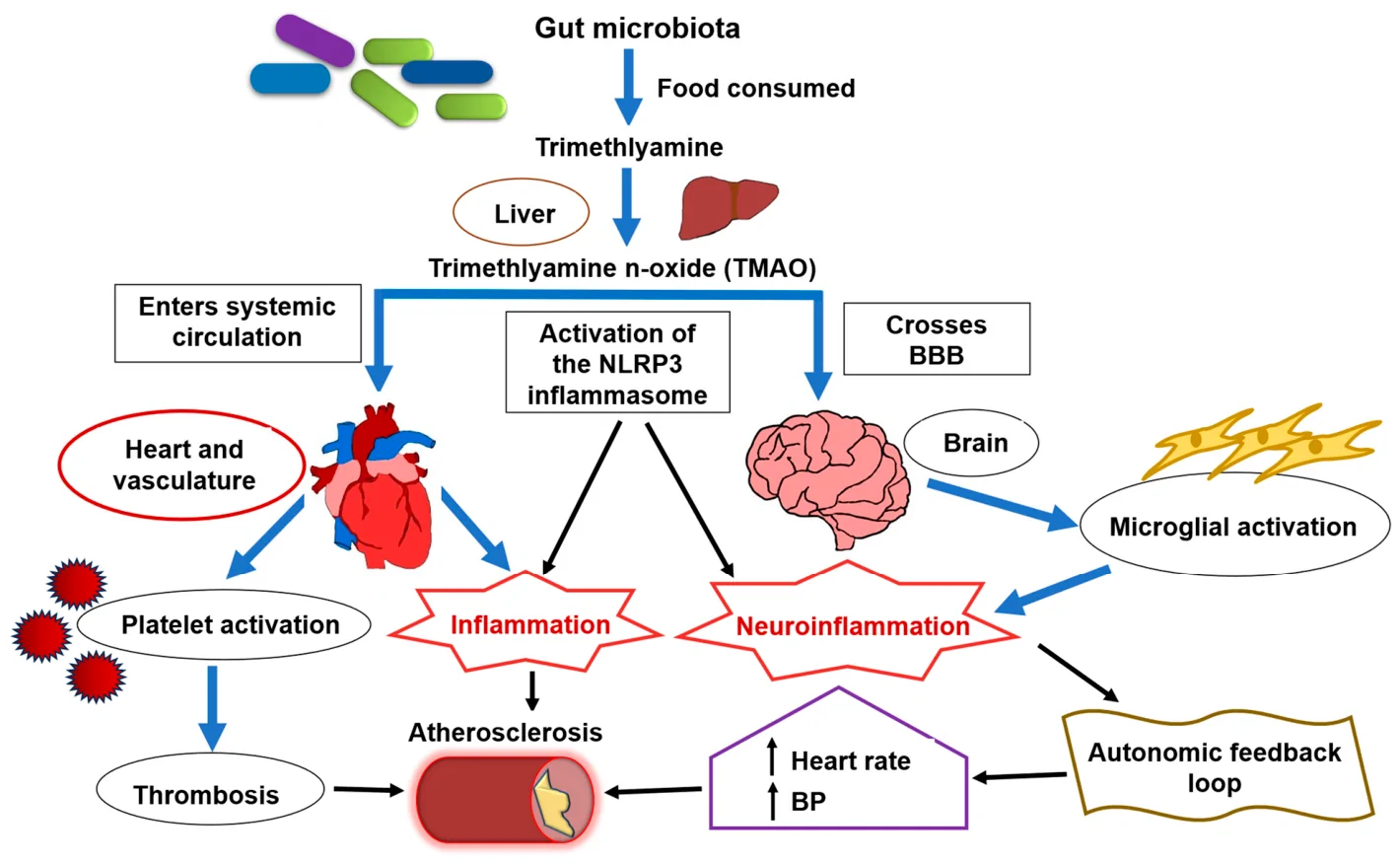
Gut microbiota and TMAO-mediated effects on heart and brain. This schematic depicts consequences of the production by gut microbiota of the dietary metabolite trimethylamine, which is subsequently converted primarily in the liver to trimethylamine-N-oxide (TMAO). TMAO promotes vascular inflammation, NLRP3 inflammasome activation, platelet-driven thrombosis, and adverse cardiac remodeling, while also disrupting autonomic regulation to amplify sympathetic activity, blood pressure, and systemic inflammation. In addition, TMAO-associated pathways contribute to neuroinflammation and microglial activation, linking gut-derived metabolites to both cardiovascular and brain dysfunction.

**Table 1:** Shared risk factors aggravated by obesity: established cardiovascular and neurovascular injury versus proposed neurodegenerative pathways.

Risk Factor	Mechanisms of Damage to the Heart	Demonstrated Mechanisms of Damage to the Brain	Proposed or Hypothetical Neurodegenerative Mechanisms
Inflammation	Accelerated atherosclerosis, cytokine release, endothelial activation, plaque formation, oxidative stress.	Neuroinflammation, microglial activation, endothelial and microvascular dysfunction, neurovascular-unit activation, BBB permeability; microglial activation, oxidative stress.	Peripheral cytokines may amplify central inflammatory signaling, impair synaptic function and interfere with neuronal energy metabolism.
Hypertension	Vascular stiffening, endothelial dysfunction, cardiomyocyte hypertrophy, increased afterload.	Microinfarcts, microbleeds, BBB dysfunction, impaired cerebral autoregulation and hypoperfusion, white-matter injury.	Chronic hypoperfusion, oxidative stress, and renin–angiotensin-system signaling may impair amyloid-β clearance and contribute to amyloid and tau pathology; the direct causal relationship remains incompletely defined.
Dyslipidemia	Lipid accumulation in the artery wall, endothelial dysfunction, oxidative damage, foam cell formation.	Microvascular injury, BBB disruption, oxidative damage, lipid toxicity, endothelial dysfunction, impaired cerebrovascular reactivity.	Altered brain lipid homeostasis, lipid peroxidation and ceramide signaling may promote amyloidogenic processing; human causal evidence remains limited and heterogeneous.
Diabetes	Metabolic stress, accelerated atherosclerosis, AGEs damage blood vessels, slowing of mitochondrial energy production, diabetic cardiomyopathy.	AGEs promote microvascular disease, reduced cerebral perfusion, white-matter hyperintensities.	Impaired brain insulin signaling, reduced amyloid-β clearance, and oxidative stress may facilitate amyloid deposition and tau hyperphosphorylation. These pathways are biologically plausible but not established.

Advanced glycation end product, AGE; blood–brain barrier, BBB.

**Table 2:** Pharmacologic strategies for cardioprotection and neuroprotection in high-risk patients.

Drug Class	Mechanisms of Risk Mitigation—Heart	Mechanisms of Risk Mitigation—Brain
Anti-hypertensives	Reduce vascular stress; preserve cardiac perfusion; lower myocardial oxygen demand and workload.	Lower blood pressure, which may impede cognitive decline and vascular dementia; preserve cerebral perfusion.
Lipid-lowering agents	Improve endothelial function; reduce myocardial infarction risk; impede atherothrombosis; lessen plaque buildup and risk of plaque rupture.	Lower ischemic stroke risk and protect cerebral vasculature. Effect on dementia risk unresolved.
Anti-inflammatory medications	Not generally cardioprotective, but colchicine is used for secondary prevention of MACE. IL-6 inhibition under study.	Not generally neuroprotective.
GLP-1 receptor agonists	Reduce the risk of MACE, even in those without diabetes. Improve cardiometabolic risk factors.	Neuroprotective potential unresolved. Reduce stroke risk in persons with diabetes.
SGLT2 inhibitors	Reduce hospitalization and cardiovascular death in patients with heart failure.	Associated with better cognitive outcomes in persons with type 2 diabetes and metabolic risk factors, but causal benefit not established.
Anti-platelet therapy	Prevents blood clots and reduces risk of myocardial infarction in patients with known cardiovascular disease.	Prevents blood clots and reduces stroke risk.

Major adverse cardiovascular events (MACE).

## Data Availability

No new data were created or analyzed in this study. Data sharing is not applicable to this article.
